# Research on the method of determining the block size for an open-pit mine integrating mining parameters and shovel-truck’s operation efficiency

**DOI:** 10.1038/s41598-024-52815-9

**Published:** 2024-05-02

**Authors:** Weiqiang Guo, Guangwei Liu, Jiaming Li, Senlin Chai, Shupeng Guo

**Affiliations:** 1 School of Mining, Liaoning Technical University, Fuxin, 123000 China; 2 School of Economics & Management, Yancheng Institute of Technology, Yancheng, 224051 China; 3 State Grid Energy Hami Coal and Electricity Co., Ltd., Dananhu No. 2 Mine, Hami, 839000 China

**Keywords:** Energy science and technology, Engineering

## Abstract

The production plan of an open-pit mine depends on the block model, so it's crucial to determine the appropriate method and size for partitioning it. This study proposes a new method based on a closed shell three-dimensional geological model for determining block model size in open-pit mines. Instead of using regular block models, the shell model is directly cut, and the discrete geological body is referred to as the "mining model." Mining parameters and the shovel-truck's performance are integrated into the method. Bench height determines the Z-axis size, bench slope angle determines the inclination angle, and shovel width determines the X-axis size of the block model. The operation efficiency of the shovel-truck considers the probability distribution of simultaneous operations, allowing the determination of the Y-axis size of block models for different types of shovels. The developed "Mining Model" module in the software "Life Cycle Mining System" is used for practical implementation. By comparing the results with traditional block models, the superiority of the proposed method is demonstrated. This study provides a more accurate model for optimizing the production plan of open-pit mines throughout their life cycle.

## Introduction

The block model serves as the foundation for various crucial tasks in open-pit mining, including production planning, grade and geological resource reserve estimation, ultimate pit design, and ore quality control. It provides a digital representation of the spatial distribution of deposit attributes. The block model's accuracy and reliability directly impact the investment, production, operational costs, and even the longevity of the mining operation^1–4^. Moreover, the open-pit mine's three-dimensional deposit model is discretized into a block model, allowing for the simulation of the dynamic evolution process throughout the mining life cycle^5,6^.

The Selective Mining Unit (SMU) represents the smallest unit in mining, designated as an ore block if the average grade of the SMU is equal to or higher than the cutoff grade; otherwise, it is considered an unminable waste block^7^. During the production phase of mining, it is theoretically ideal for the block size to align with the SMU, ensuring that the extracted tonnage closely approximates the results of reserve estimation. Therefore, the rational selection of block size in resource reserve estimation is essentially a determination of the SMU. In the case of open-pit mining, the determination of the SMU is influenced by various factors, categorized into geological, technical, and economic factors. Technical factors include mining parameters and equipment operational capabilities specific to open-pit mining.

The choice of block size and shape holds significant importance in resource quality assessment, mine optimization, and mine planning processes, directly influencing the economic viability of mining projects. Intuitively, a smaller block size should result in a more accurate block model. However, during practical implementation, the statistical problems of geological spatial information, and computer computing power constraints pose challenges in determining the optimal block size. Nevertheless, establishing the appropriate block size remains a crucial factor for effective open-pit mine production scheduling and the optimization of mining operations^8^. Currently, the prevailing approach in open-pit mining involves the utilization of the conventional block model construction method. This method entails filling the three-dimensional geological solid model with standardized blocks of a set size. Through this process, the construction of the block model is accomplished, and the three-dimensional geological model is discretized accordingly^9^. Subsequently, the spatial estimation interpolation method is employed to assign the geological attributes to each individual block, thereby facilitating the simulation of open-pit mining operations based on the block's designated mining sequence^10,11^.

The determination of block size division parameters is a complex engineering decision that has been extensively studied by numerous scholars. Yarahmadi^12^ developed a computer program to determine geometry and size of rock blocks in two dimensional spaces. Hartman^13^ emphasized that the size of the block model can be determined by considering the mining method and the selectivity of the ore, aiming to minimize ore dilution. David^14^ conducted a comprehensive study on the block size problem utilizing advanced geostatistical methods. The research highlighted the crucial significance of block size selection in determining recoverable resource reserves, evaluating resource quality, optimizing mine designs, and formulating mining plans. The findings emphasized that the choice of block size directly impacts the economic benefits of mining operations. Jara et al.^15^ also conducted a study investigating the impact of block model size on open-pit mine design and production planning. They quantified the influence of block model size on mining selectivity, such as grade and dilution, and assessed its effects on the economic outcomes of mining, including income, costs, and discounted cash flow. The findings revealed that as the block size increases, the overall quality of the ore body improves, but the average grade decreases. Additionally, the study observed a decrease in discounted cash flow with larger block model sizes. Similarly, Birch^16^ examined the influence of block size on the final grade-tonnage curve and observed its impact on the average grade of the ore body. Furthermore, variations in block size were found to affect the overall income and net present value of the mine. Ruiseco^17^ proposed guidelines for determining the block size in relation to the horizontal direction of the ore body. The recommendation suggests that the block size should be greater than 1/4 of the average distance between exploration boreholes and not less than 1/3 of the distance between two boreholes. Furthermore, it is advised that the block height should not exceed the height of the working bench. Furthermore, several researchers have taken into account various uncertainties, including average grade, recovery rate, production capacity, and dilution degree. They have employed Multi-Criteria Decision-Making (MCDM) methods to effectively select the appropriate size of the block model. Leuangthong et al.^18,19^ highlighted that the determination of block size relies on various factors, including mining equipment, mining method, mining direction, and the sedimentary environment of the ore body. Recognizing the interconnected nature of these factors, they proposed the utilization of the Analytic Hierarchy Process (AHP) to select the optimal block size for mine production planning. Hayati et al.^8^ employed the VlseKriterijumska Optimizacija I Kompromisno Resenje (VIKOR) method to ascertain the optimal block size for the Angouran open-pit mine in Iran, resulting in the determination of a 10 m-sized block model. Subsequently, Abdollahei^20^ proposed the utilization of the Fuzzy Delphi Analytic Hierarchy Process (FDAHP) and Fuzzy Multi-Objective Ratio Analysis (FMOORA) to optimize the size of the block model. These methods provide a framework for assessing and determining the most favorable block model size. Stevanovic et al.^21^ adopted a range of 1/3 to 1/2 of the borehole spacing in the *X* and *Y* directions, based on the arrangement of the exploration work, as the division size range for blocks. The *Z* direction was constrained by the bench height, determining multiple sets of block size division schemes. Ultimately, the (AHP) was utilized to identify the optimal block size for mine production planning, considering the defined range of division sizes.

In summary, researchers have dedicated their efforts to studying the impact of block size on ore dilution and boundary grade, as well as determining the most suitable block size to minimize ore dilution and error in boundary grade selection. In theory, reducing the block size leads to improved accuracy and minimizes the effects on ore dilution and boundary grade. However, practical limitations in computer computing power prevent achieving the ideal block size. It is essential to acknowledge that no matter how small the block size becomes, it can only approximate the original three-dimensional geological body and cannot fully represent the volume and grade of the actual entity. Simultaneously, researchers commonly employ techniques such as integer programming, linear programming, mixed integer programming, and dynamic programming to formulate the mathematical model for open-pit mine production planning^22–25^. The mathematical models developed using the methods rely on regular cube or cuboid blocks as the foundational representation. However, during the simulation of mine production, the shape of the working bench side often deviates from the regular shape observed in practical mining operations. Meanwhile, the formulation of the open-pit mine production plan does not accurately correspond to the real output of each excavator's working area as depicted in the block model. Therefore, this research plan has introduced a novel approach by altering the foundation of the block model. It proposes a method to divide the size of the open-pit mine block model, taking into account the stripping process parameters and the efficiency of vehicle-shovel equipment.

Open-pit coal mining employs benches as the fundamental mining operational unit, extracting in a strip-sequence manner. The mining parameters and the operational capabilities of the truck-shovel system are inherently related to the block model. Thus, this study departs from the conventional approach of approximating the three-dimensional geological model using regular block models to represent the mining process, instead mapping the stripping parameters and equipment operational capabilities onto the block model. This serves as a dimensional parameter for block model segmentation. This approach can reduce constraints in subsequent studies on optimizing open-pit coal mining plans, enhance the efficiency of solving optimization models, and align more closely with on-site construction and operational realities.

Based on this research approach and considering temporary equipment failures, maintenance, and unbalanced truck-shovel combinations in actual production operations that affect the overall production system output, thereby influencing the practical operational capabilities of the truck-shovel system, the study incorporates a probabilistic analysis method as presented in reference^26^. The literature suggests that the actual number of operational trucks and shovels fluctuates around a certain mathematical expectation, and the study employs probability analysis to investigate the actual operational capabilities of the equipment. Each mining equipment's daily production is considered the volume of an individual mining block in the field, integrating mining parameters. The study proposes a method that integrates mining parameters and truck-shovel equipment operational capabilities into the discretization of a three-dimensional geological model for open-pit coal mining. This approach provides a crucial model foundation for optimizing production plans in open-pit coal mining.

## Mining parameters constraint

**Figure 1:**
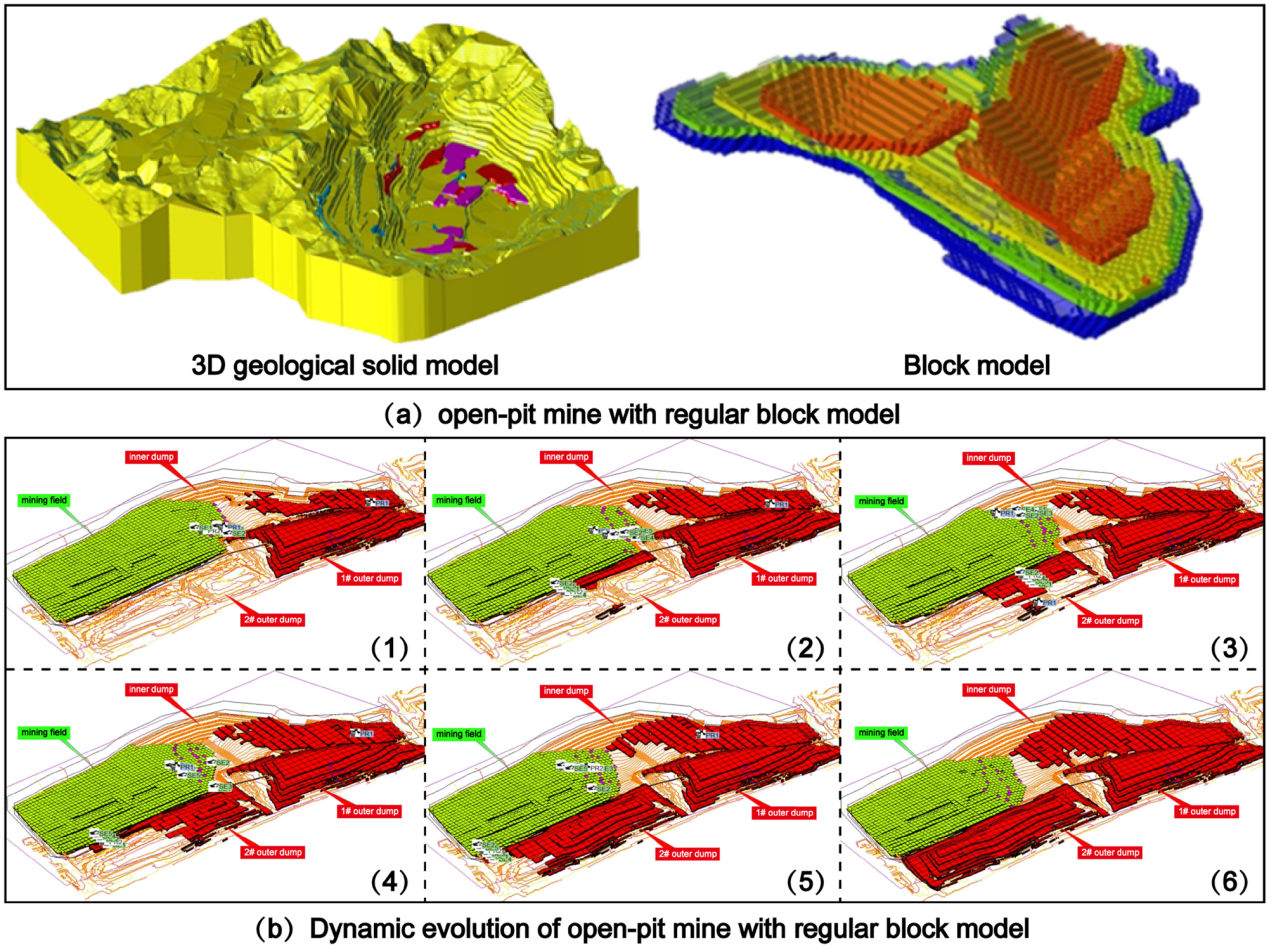
Dynamic evolution of the open-pit mine production scheduling with regular block models.

**Figure 2:**
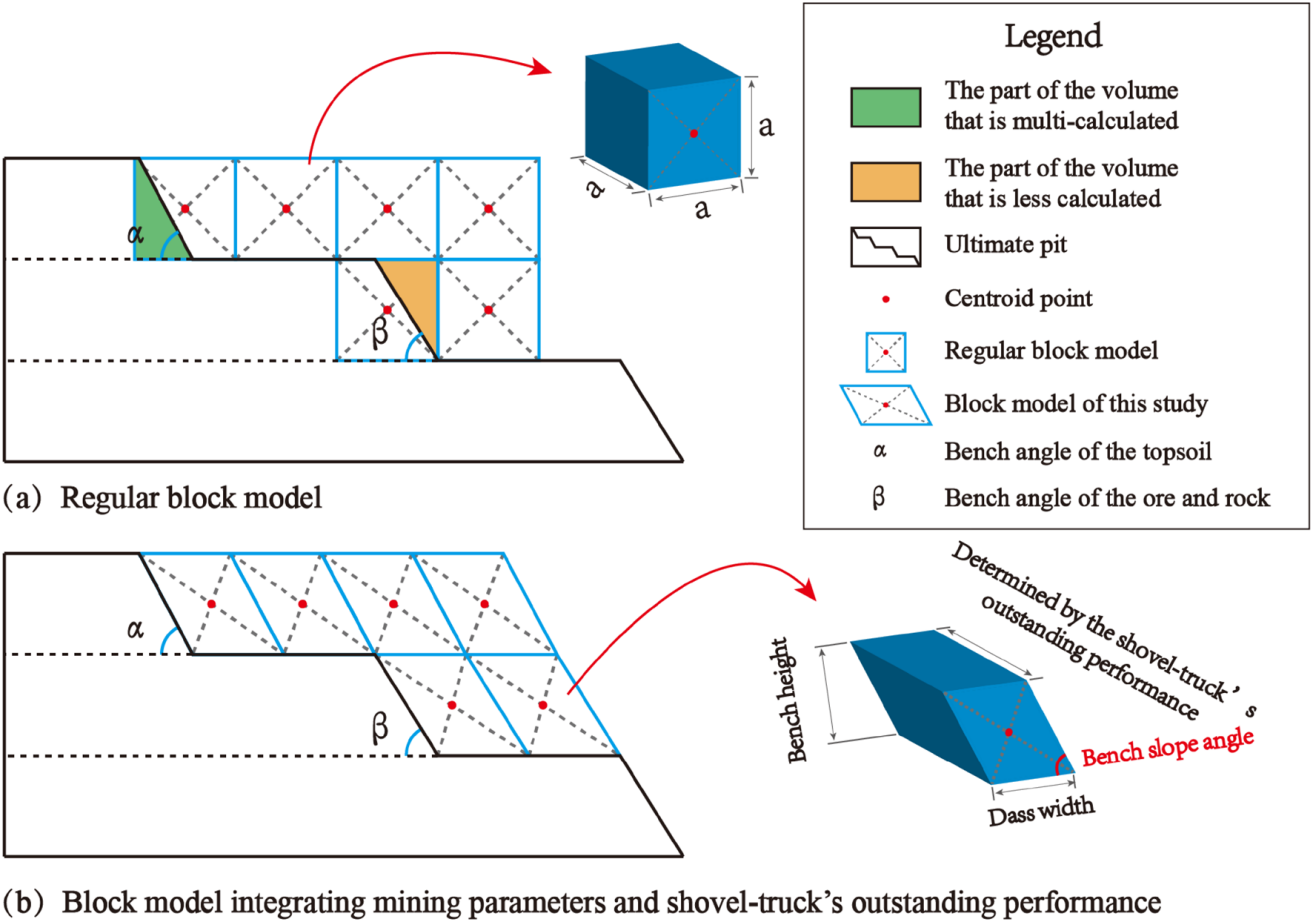
Comparison of ore deposit block models.

**Figure 3:**
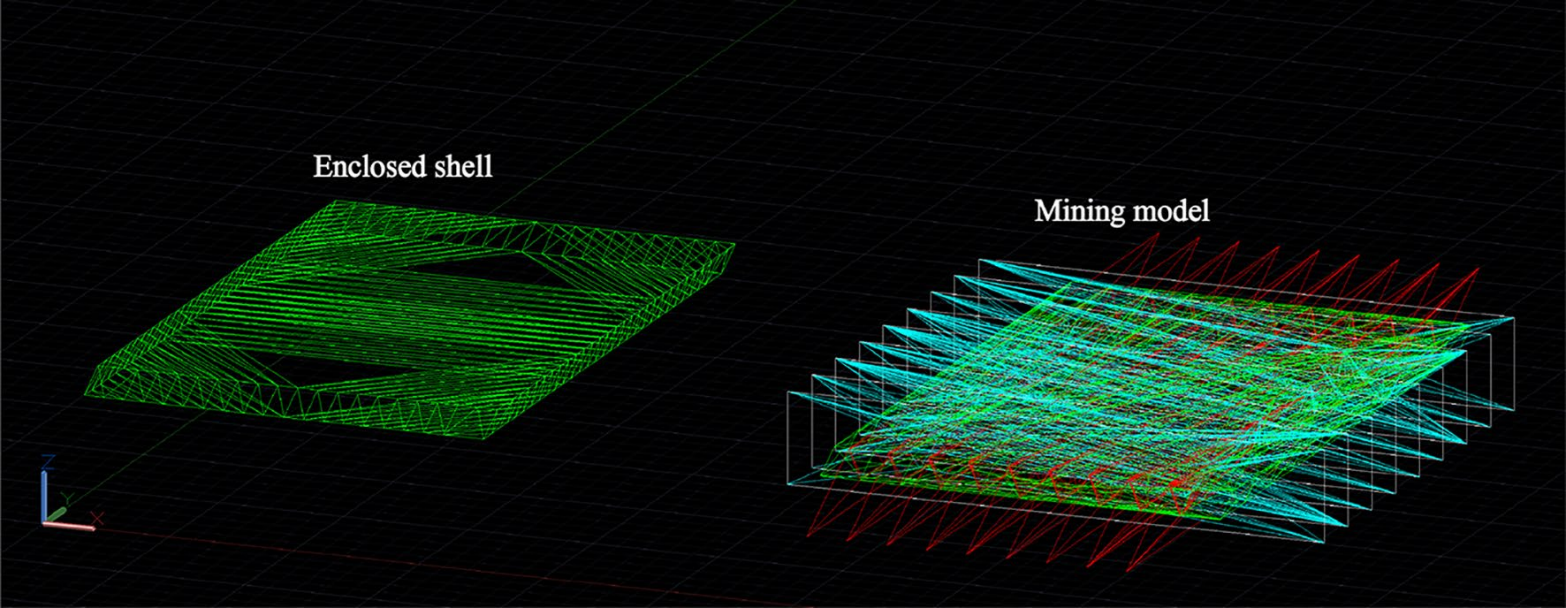
Mining model.

The conventional approach involves utilizing the standard block model to populate the three-dimensional geological body. Subsequently, the dynamic deduction of the open-pit mine production plan is carried out from this foundation, as depicted in Fig. 1. When the block model is filled to the irregular final pit boundary, if the centroid position of the block is within the boundary, the block is filled, but there will be a multi-volume calculation part. If the mass center point of the block is outside the boundary, it will not be filled, but the volume will be undercounted, as shown in Fig. 2a. In cases where the geological body is extensive, and there exist thousands of block models, the cumulative effect of errors arising from block filling at the final pit boundary becomes significant. This, in turn, adversely impacts the accuracy of determining the quantity of ore and rock during the formulation of the production plan. The block model proposed in this study is generated by cutting directly on the closed shell 3D geological body, as shown in Fig. 3, and the discrete geological body is defined as ‘Mining model’.

The mining parameters depend on the open-pit mining technology. The basic parameters include bench height *h*, bench slope angle *α*, mining belt width *A*, and bench width *B*^27^. Taking into consideration the on-site operations, the constrained block model incorporates the actual dimensions. The *Z*-axis size of the block corresponds to the bench height *h*. The *Y*-axis size represents the mining belt width *A*. The block dip angle is equivalent to the bench slope angle *α*. Lastly, the *X*-axis size is determined based on the daily operation efficiency of various excavator types. The division method proposed in this paper has no deviation from the three-dimensional geological body in accuracy and ensures the integrity of the step shape. The specific morphology is shown in Fig. 2b.

### Bench height constraint

The ore and rock in open-pit mines is generally divided into multiple benches for extraction. The division of benches should be conducive to maximizing equipment efficiency, improving ore quality, and ensuring operational safety. The bench height is one of the most important geometric parameters in open-pit mining^28^. Factors influencing the bench height include the burial conditions and properties of rock formations, operational conditions of mining equipment, drilling and blasting operations, production scale, technical specifications of mining and loading equipment, layout of transportation routes, and mining advancing speed, among others. For excavators with a bucket capacity of 3 m^3^ to 4 m^3^, the bench height ranges from 10 to 15 m. Larger excavators can have bench heights of up to 20 m to 25 m, while large recasting excavators can reach bench heights of over 30 m. Wheel and chain excavators can have bench heights of 40 m to 50 m. Therefore, the selection of bench height should be determined based on the type of excavator.

### Bench slope angle constraint

The bench slope angle primarily depends on the stability of the rock mass, and its value increases as the rock mass stability improves. Furthermore, the inclination of the bedding plane in the rock mass directly affects the bench slope angle. When the inclination of the bedding plane aligns or closely resembles that of the bench slope, and the bedding plane has a steep dip angle, the bench slope angle equals the inclination of the bedding plane.

### Mining belt width constraint

In mining operations, the benches are typically divided into sequential strips of certain widths based on the direction of rock layers, known as mining belt. The mining belt width represents the width of excavation carried out by the excavator in one pass, and it influences various aspects such as drilling and blasting, mining and loading, track relocation, and bench width. Consequently, the mining belt width plays a crucial role in determining the progress of stripping and mining operations. In cases where the ore and rock are soft and do not require blasting, the mining area width is equivalent to the mining belt width. However, for ore and rock that necessitate blasting, the mining belt width generally refers to the width of one mining operation after a single blast, commonly known as "one blasting, two mining" or "one blasting, one mining". The determination of the mining belt width is primarily based on the technical specifications of the excavator. It is important to ensure that the mining belt width does not exceed 1.5 times the radius of the maximum excavator at the standing level, denoted as *A* ≤ 1.5 *R*_*wp*_. Additionally, the minimum width of the mining belt should meet the following requirements:

1 $$A_{\min } \ge h\cot \alpha + e,m$$

where $e$ is the safe distance from the center line of the drilling rig to the top line of the bench slope, generally 2.5–3 m; $h$ is bench height, m; $\alpha$ is bench slope angle, °.

When using track transportation, due to its flexibility, small turning radius, and climbing ability, it can effectively cooperate with the mining equipment in the production to improve the mining efficiency. Simultaneously, the mining belt width can be set without considering the problem of blasting pile buried road after blasting. The mining belt width can be larger, which is only related to the technical specifications of the excavator, the shunting mode and the safe distance of the transportation channel.

## Shovel-truck’s operation efficiency constraint

Equipment operation efficiency refers to the ability of equipment to achieve maximum production efficiency with minimal resource consumption, while ensuring quality during the production process^29,30^. In this study, the analysis and calculation will be carried out using the example of the coordinated operation between open-pit mining shovels and trucks. Furthermore, to align with practical applications, the matching operation of multiple equipment models between self-operated and outsourcing units is taken into consideration.

In a multi-link production system, the equipment within each production link is operated in a sequential and mutually constrained manner. The production system consists of *M* production links. The *i* link contains *N*_*i*_ equipment with an average operation rate of *p*_*i*_. The actual number of units in the system will fluctuate around a specific mathematical expectation, causing corresponding changes in the overall system index. Probability analysis can be employed to study this distribution characteristic, facilitating the rational selection and utilization of equipment.

### Probability distribution of simultaneous operation equipment quantity

The self-operated and outsourced units within the system collectively possess *x* devices, and the probability of simultaneous operation follows a binomial distribution:

2 $$P\left( x \right) = C_{N}^{x} p^{x} \left( {1 - p} \right)^{N - x} = \frac{N!}{{x!\left( {N - x} \right)!}} \cdot \left( {1 - p} \right)^{N - x}$$

where *N* is the total number of equipment, *N*_*S*_ denotes the number of shovels, and *N*_*T*_ represents the number of trucks; *x* is the number of devices operating simultaneously; *p* is the average operating rate of the equipment, *p*_*s*_ is the average operating rate of the shovels, and *p*_*t*_ is the average operating rate of the trucks.

For convenience, the following recursive formula can be utilized to calculate the probability values for different *x*:

3 $$P\left( {x + 1} \right) = \frac{N - x}{{x + 1}} \cdot \frac{p}{1 - p} \cdot P\left( x \right)$$

From Eq. (3), the probability distribution matrix of the number of shovel-truck simultaneous operations can be obtained. Among them:

4 $$P\left( {i,j} \right) = P_{s} \left( i \right) \cdot P_{t} \left( j \right)$$

where $P\left( {i,j} \right)$ is the probability of simultaneous with *i* shovels and *j* trucks; $P_{s} \left( i \right)$ is the probability of *i* shovels operating simultaneously; $P_{t} \left( j \right)$ is the probability of *j* trucks operating simultaneously.

Assuming the system consists of: shovel *N*_*s*_ = 1 and truck *N*_*T*_ = 5. The shovel operation rate is *p*_*s*_ = 0.8, and the truck operation rate is *p*_*t*_ = 0.75. The probability distribution of simultaneous shovel-truck operations can be found in Table 1.

**Table 1:** Probability distribution of simultaneous operation equipment quantity $\left( {N_{s} = 1,N_{t} = 5,p_{s} = 0.8,p_{t} = 0.75,k_{0} = 4} \right)$.

_Operation truck number_	^Operation shovel number^	
	0	1
0	**0.0001953**	**0.0007813**
1	**0.0029298**	0.0117192
2	**0.0175788**	0.0703152
3	**0.0527364**	0.2109156
4	**0.0791046**	0.3164184
5	**0.0474628**	*0.1898510*

Bold: No product area.

Italics: Oversaturated area.

By conducting an analysis of Table 1, it becomes evident that the probability matrix can be effectively partitioned into three distinct regions:

1. No product area. That is, the number of working shovels or the trucks is zero.
2. Oversaturated area. It refers to the area where the truck to shovel ratio is too large and the truck cannot give full play to its efficiency. When the quantities of trucks and shovels are not zero and the shovel quantity is fixed, the probability values gradually increase as the number of trucks grows. Once a certain quantity is reached, the probability values start to decrease. The region where the probability values decrease is defined as the oversaturated area.
  The research findings indicate that Fig. 4 illustrates the correlation between the efficiency of each shovel or truck and the truck to shovel ratio. When the truck to shovel ratio *K* is below a critical value *K*_0_, the efficiency *Q*_*s*_ of each shovel demonstrates a proportional relationship with the truck to shovel ratio *K*. However, once *K* > *K*_0_, the growth rate of *Q*_*s*_ becomes significantly sluggish. The efficiency *Q*_*t*_ of each truck primarily remains constant when *K* < *K*_0_. However, when *K* > *K*_0_, the efficiency *Q*_*t*_ becomes inversely proportional to *K*, indicating that as *K* increases, the efficiency of each truck decreases. The region where *K* > *K*_0_ is referred to as the oversaturated area, signifying an excessive number of trucks that prevent the improvement of shovel efficiency and lead to a decrease in truck efficiency. This ultimately results in wasteful utilization of equipment. In Table 1, the division is made based on *K*_0_ = 4 to delineate the different areas.

**Figure 4:**
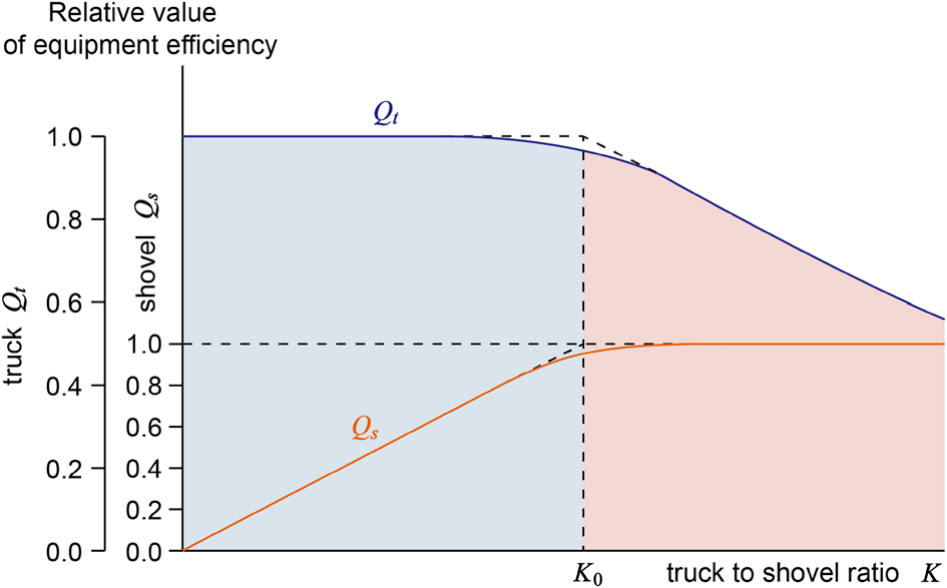
Relationship between efficiency of truck and shovel and truck to shovel ratio.
3. General operating area: This classification encompasses the regions that are not part of the previously mentioned no product area and oversaturated area.

### System production capacity and equipment efficiency

The total system production capacity can be obtained by Eq. (5):

5 $$Q_{c} = \sum\limits_{i = 1}^{{N_{s} }} {\sum\limits_{i = 1}^{{N_{t} }} {P\left( {i,j} \right) \cdot Q\left( {i,j} \right)} }$$

where *Q*_*c*_ is the total system production capacity; *P*(*i*, *j*) is the probability of *i* shovels and *j* trucks operating simultaneously; *Q*(*i*, *j*) is the system production capacity of *i* shovels and *j* trucks operating simultaneously; *N*_*s*_ is the number of shovels in the system; *N*_*t*_ is the number of trucks in the system.

Considering the simultaneous operation of both self-operated and outsourced equipment, a combination of various types of shovel and truck equipment is employed to work together. As a result, the total system production capacity can be determined:

6 $$Q_{c} = \sum\limits_{m = 1}^{M} {Q_{m} }$$

7 $$Q_{m} = \sum\limits_{i = 1}^{{N_{s} }} {\sum\limits_{j = 1}^{{N_{t} }} {P\left( {i,j} \right) \cdot N_{t}^{\prime} \cdot Q_{0} } } = \sum\limits_{i = 1}^{{N_{s} }} {\sum\limits_{j = 1}^{{iK_{0} }} {P\left( {i,j} \right) \cdot j \cdot Q_{0} } } + \sum\limits_{i = 1}^{{N_{s} }} {\sum\limits_{{j = iK_{0} + 1}}^{{N_{t} }} {P\left( {i,j} \right) \cdot iK_{0} \cdot Q_{0} } }$$

where *Q*_*m*_ is the production capacity of each set of shovel-truck; $N_{t}^{\prime}$ is the number of trucks calculated, when $j \le iK_{0}$, $N_{t}^{\prime} = j$, when $j > iK_{0}$, $N_{t}^{\prime} = iK_{0}$; *Q*_0_ is the efficiency of each truck when shovels and trucks are working continuously; *K*_0_ is the truck to shovel ratio saturation value (round-off number).

The average efficiency of each equipment is:

8 $$Q_{s} = \frac{{Q_{c} }}{{N_{s} }}$$

9 $$Q_{t} = \frac{{Q_{c} }}{{N_{t} }}$$

where *Q*_*s*_ is the average efficiency per shovel; *Q*_*t*_ is the average efficiency per truck.

Suppose $Q_{0} = 10^{6}$ t/a, $K_{0} = 4$,the following results are obtained.

When $$N_{s} = 1$$, $$N_{t} = 5$$, $$Q_{c} = 2.810264 \times 10^{6}$$ t/a; $$Q_{s} = 2.810264 \times 10^{6}$$ t/a; $$Q_{t} = 5.62053 \times 10^{5}$$ t/a.

## Block model size

The volume $$V$$ of a single block is determined by the daily production capacity of a shovel. The number of working days per year is calculated at 330. Once the mining technology and operational equipment model are determined, the bench height *h*, the bench slope angle *α*, and the bench width *A*, that is, *Z*-axis size $$\overset{\lower0.5em\hbox{$\smash{\scriptscriptstyle\frown}$}}{Z}$$ and *Y*-axis size $$\overset{\lower0.5em\hbox{$\smash{\scriptscriptstyle\frown}$}}{Y}$$ of the block model can be determined. With these values known, the *X*-axis size $$\overset{\lower0.5em\hbox{$\smash{\scriptscriptstyle\frown}$}}{X}$$ can be obtained using the volume calculation Eqs. (10) and (11).

10 $$V = \overset{\lower0.5em\hbox{$\smash{\scriptscriptstyle\frown}$}}{X} \cdot \overset{\lower0.5em\hbox{$\smash{\scriptscriptstyle\frown}$}}{Y} \cdot \overset{\lower0.5em\hbox{$\smash{\scriptscriptstyle\frown}$}}{Z}$$

11 $$\overset{\lower0.5em\hbox{$\smash{\scriptscriptstyle\frown}$}}{X} = \frac{V}{{\overset{\lower0.5em\hbox{$\smash{\scriptscriptstyle\frown}$}}{Y} \cdot \overset{\lower0.5em\hbox{$\smash{\scriptscriptstyle\frown}$}}{Z} }} = \frac{{Q_{s} }}{330 \cdot A \cdot h} = \frac{{\tilde{Q}}}{A \cdot h}$$

where $\tilde{Q}$ is the shovel’s daily operation efficiency.

**Figure 5:**
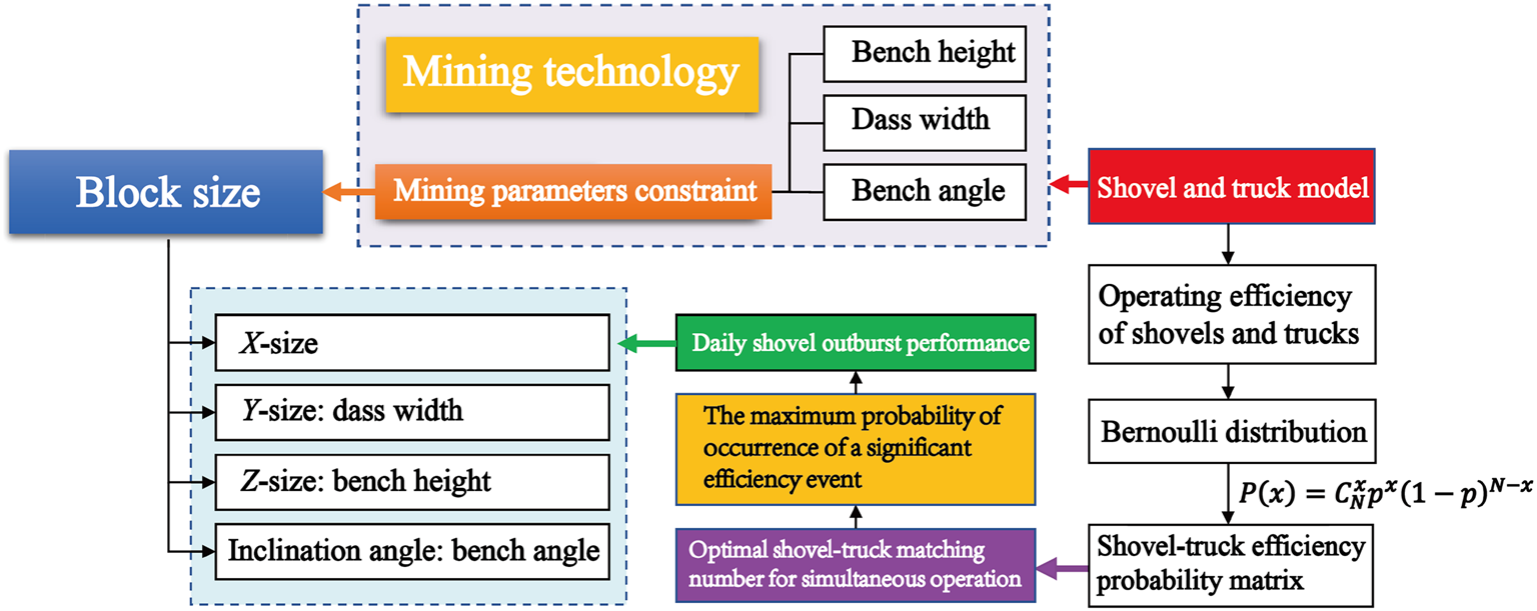
Flow chart of the block model size determination method.

The specific process of the method proposed in this study is depicted in Fig. 5.

## Case study

**Figure 6:**
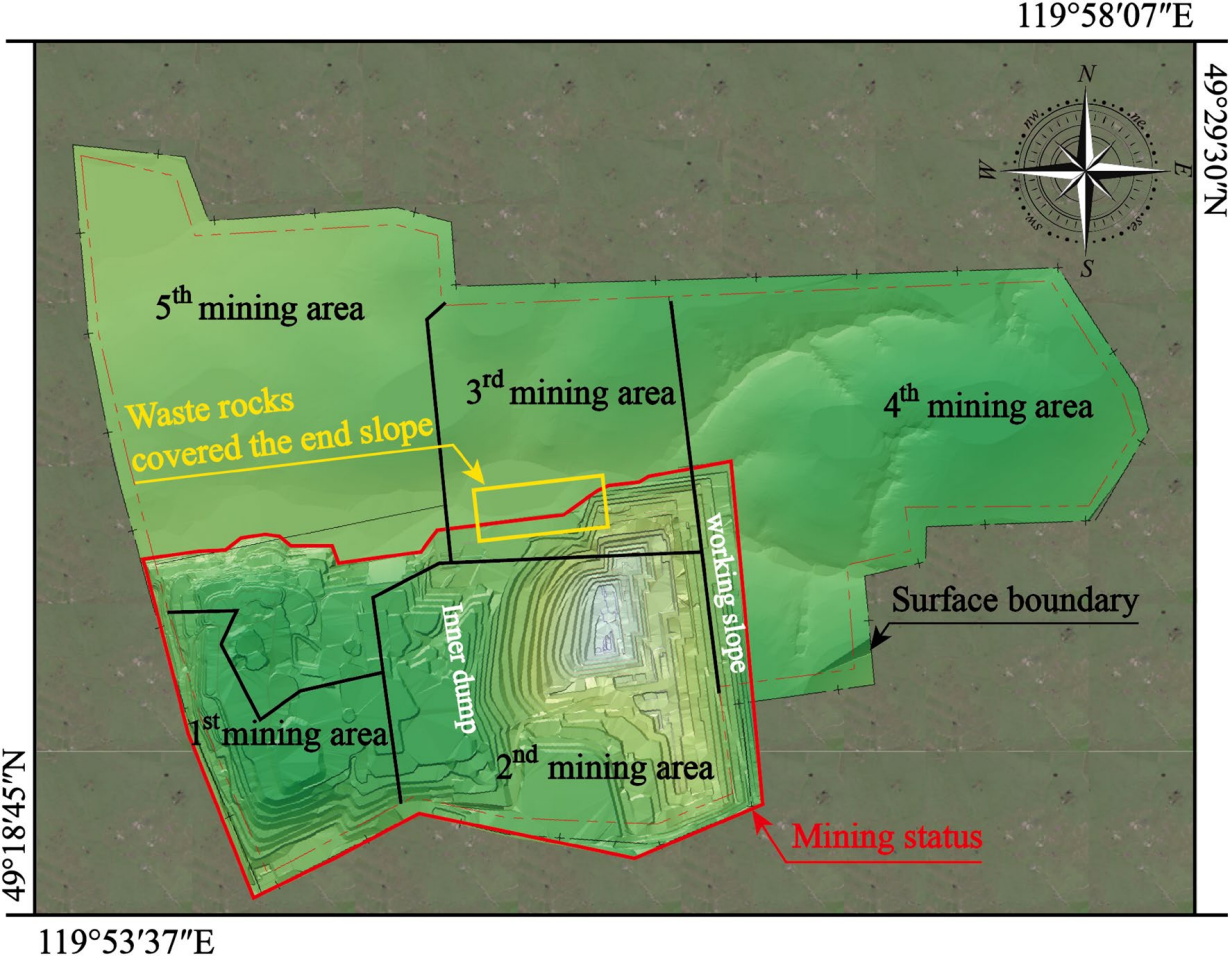
The Baorixile open-pit mine.

This study utilizes the Baorixile open-pit mine as a case study (as shown in Fig. 6). The Chenbaerhuqi coal field in Hulunbuir City, Inner Mongolia Autonomous Region of China, is where the Baorixile open-pit coal mine is situated^31,32^. The mine's external perimeter spans a 50.72 km^2^ region, measuring 5.86 km in width from north to south and 10.98 km in length from east to west. The mine was constructed in 1998. The primary coal seams targeted for mining are coal B, coal 1^2^, coal 2^1^, and coal 3^1^. The original design’s annual production capacity was 1.80 million t. In 2014, the approved production capacity of the Baorixile Open-pit Coal Mine was 35 million t/a. The semi-continuous mining technology of shovel-single bucket truck-semi-fixed crushing station is adopted.

The self-operated and outsourcing units’ shovels and trucks of the open-pit mine simultaneously performs on-site coal mining and rock stripping operations^33^. The models of self-operated trucks and shovel are as follows:

1. Coal mining equipment models
  The single bucket excavators of WK-35 (bucket capacity 35 m^3^), WK-10B (bucket capacity 10 m^3^) and WK-12 (bucket capacity 12 m^3^) are selected for the coal mining equipment, and the dump trucks of MT4400AC-220T (load 220t) and TR100 (load 91t) are selected for the coal transportation equipment.
2. Rock stripping equipment models
  The single bucket excavators of WK-35 (bucket capacity 35 m^3^), WK-10B (bucket capacity 10 m^3^) and WK-12 (bucket capacity 12 m^3^) are selected for rock stripping equipment, and the dump trucks of MT4400AC-220T (load 220t) and TR100 (load 91t) are selected for rock transportation equipment.
  The outsourcing equipment is mainly responsible for rock stripping, and their models are 4 m^3^ hydraulic shovel and 60t dump truck.
  Table 2 presents the models, quantities, and operational efficiencies of all shovels and trucks utilized in the open-pit mine.

**Table 2:** Equipment models and quantities.

Equipment		Model	Quantity	Operating efficiency
Self-management	Shovel	WK-35	6	0.96
		WK-10B	4	0.95
		WK-12	2	0.92
	Truck	MT4400AC-220T	30	0.93
		TR100	34	0.94
Outsourcing	Shovel	4 m^3^ hydraulic shovel	113	0.93
	Truck	60t dump truck	365	0.80

### Block model

#### Mining parameter

The following mining parameters are obtained from the preliminary design report of the mine^33^.

1. Bench height
  The bench height is determined by considering the properties of the stripping soil and rock, process characteristics, equipment specifications, mining requirements, and a comprehensive assessment aimed at enhancing equipment operating conditions and improving production efficiency^34^. Bench height stands as a significant mining parameter in open-pit mines. To determine the appropriate bench height for stripping and coal mining operations, several factors are considered, including the maximum excavation height of hydraulic excavators, production stripping ratio, and other relevant considerations.
  Based on a careful evaluation of the physical and mechanical properties of the stripping material and its burial conditions, as well as considering the specifications of the mining equipment, the determined stripping step height for the mine is 15 m (that is $$\overset{\lower0.5em\hbox{$\smash{\scriptscriptstyle\frown}$}}{Z}_{r}$$ = 15 m). The benches are horizontally divided to ensure effective and efficient mining operations.
  The coal seam of this mine is a nearly horizontal and the dip angle is 1–3°. Hence, the standard coal bench height is set at 15 m, denoted as $$\overset{\lower0.5em\hbox{$\smash{\scriptscriptstyle\frown}$}}{Z}_{c}$$ = 15 m.
2. Bench slope angle
  The topsoil bench slope angle is 65°, that is α_*t*_ = 65°; the coal and rock bench slope angle are 70°, that is α_*c*_ = 70°, α_*r*_ = 70°.
3. Mining belt width
  Based on process characteristics, it is observed that a wider mining belt width leads to a reduced number of pit lines in the annual operation, resulting in higher system efficiency. However, an increase in the mining belt width will result in a slower working slope angle, leading to an increase in the amount of stripping work required. Considering the specifications of the working equipment, mining and loading conditions, and other relevant factors, the mining belt width for each stripping equipment is determined as follows:
  ① The mining belt width of shovel WK-35 is 25 m, that is $$\overset{\lower0.5em\hbox{$\smash{\scriptscriptstyle\frown}$}}{Y}_{WK - 35} = 25$$ m;
  ② The bench width of shovel WK-10B is 20 m, that is $$\overset{\lower0.5em\hbox{$\smash{\scriptscriptstyle\frown}$}}{Y}_{WK - 10B} = 20$$ m;
  ③ The bench width of shovel WK-12 is 20 m, that is $$\overset{\lower0.5em\hbox{$\smash{\scriptscriptstyle\frown}$}}{Y}_{WK - 12} = 20$$ m;
  ④ The mining belt width of 4 m^3^ hydraulic backhoe is 10 m, that is $$\overset{\lower0.5em\hbox{$\smash{\scriptscriptstyle\frown}$}}{Y}_{{4m^{3} }} = 10$$ m.

#### Shovel-truck’s operation efficiency

Table 2 illustrates the operational efficiency of various shovel types in both self-operated and outsourcing units as follows: *P*_WK-35_ = 0.96, *P*_WK-10B_ = 0.95, *P*_WK-12_ = 0.92, *P*_4m3_ = 0.93. Operating efficiency of different types of trucks: *P*_MT4400AC-220 T_ = 0.93, *P*_TR100_ = 0.94, *P*_60t_ = 0.80.

Based on Eq. (3), the probability distribution of the number of shovels and trucks operating simultaneously can be calculated, as demonstrated in Table 3.

**Table 3:** Probability distribution of the number of shovels and trucks operating simultaneously at the Baorixile open-pit mine.

Number	Shovel model				Truck model		
	*P*_WK-35_	*P*_WK-10B_	*P*_WK-12_	*P*_4m3_	*P*_MT4400AC-220T_	*P*_TR100_	*P*_60t_
0	4.0960E−09	6.2500E−06	6.4000E−03	3.1339E−131	2.2539E−35	2.8651E−42	1.8607E−319
1	5.8982E−07	4.7500E−04	1.4720E−01	4.7048E−128	8.9835E−33	1.5262E−39	3.3939E−316
2	3.5389E−05	1.3538E−02	8.4640E−01	3.5004E−125	1.7306E−30	3.9451E−37	3.0884E−313
3	1.1325E−03	1.7148E−01		1.7207E−122	2.1460E−28	6.5927E−35	1.8695E−310
4	2.0384E−02	8.1451E−01		6.2866E−120	1.9245E−26	8.0046E−33	8.4699E−308
5	1.9569E−01			1.8208E−117	1.3295E−24	7.5244E−31	3.0627E−305
6	7.8276E−01			4.3543E−115	7.3599E−23	5.6976E−29	9.2085E−303
7				8.8428E−113	3.3525E−21	3.5705E−27	2.3679E−300
8				1.5566E−110	1.2805E−19	1.8879E−25	5.3159E−298
9				2.4128E−108	4.1587E−18	8.5445E−24	1.0585E−295
10				3.3338E−106	1.1603E−16	3.3466E−22	1.8926E−293
…				…	…	…	…

Due to the extensive volume of data, only a portion of the data is presented in Table 3. Based on the data calculation presented in Table 3, it can be concluded that the maximum probability of simultaneous operation for all self-operated and outsourced equipment is *P* = 2.8483E−04. Then the combination of utilizing 6 WK-35, 4 WK-10B, 2 WK-12, 28 MT4400AC-220T, 32 TR100, 106 4 m^3^ hydraulic shovels, and 365 60t dump trucks exhibits the highest probability, as depicted in Table 4. Simultaneously, Table 4 showcases the specific combinations of trucks and shovels employed in the actual production operations of the open-pit mine.

**Table 4:** Truck to shovel ratio and the number of matching truck-shovel.

Operatin tupes	Shovel-truck combination	The efficiency of each type of truck when the shovel and the truck are continuously operated (10^4^ m^3^/a)	Truck to shovel ratio saturation value *K*_0_	Number of excavating equipment				Number of transport equipment		
				WK-35	WK-10B	WK-12	4 m^3^ hydraulic backhoe	MT4400AC-220T	TR100T	60t dump truck
Self-operation	WK-35 + MT4400AC-220T	115	5	6	–	–	–	28	–	–
	WK-10B + TR100T	61	5	–	4	–	–	–	20	–
	WK-12 + TR100T	61	5	–	–	2	–	–	12	–
Outsourcing	4 m^3^ hydraulic backhoe + 60t dump truck	40	3	–	–	–	106	–	–	365
Summary	–	–	–	6	4	2	106	28	32	365

According to the specific saturation value *K*_0_ of each combination of trucks and shovels in Table 4, as well as the working efficiency of each type of truck when both the shovel and the truck are continuously operated, the production capacity of each combination can be calculated using Eq. (7) as follows:

① WK-35 + MT4400AC-220T:
  $$Q_{WK - 35} = \sum\limits_{i = 1}^{{N_{s} }} {\sum\limits_{j = 1}^{{iK_{0} }} {P\left( {i,j} \right) \cdot j \cdot Q_{MT400AC - 200T} } } = 1954.16 \cdot {1}0^{{4}} \;{\text{m}}^{{3}} {\text{/a}}$$
② WK-10B + TR100T:
  $$Q_{WK - 10B} = \sum\limits_{i = 1}^{{N_{s} }} {\sum\limits_{j = 1}^{{iK_{0} }} {P\left( {i,j} \right) \cdot j \cdot Q_{TR100T} } } = 993.21 \cdot {1}0^{{4}} \;{\text{m}}^{{3}} {\text{/a}}$$
③ WK-12 + TR100T:
  $$Q_{WK - 12} = \sum\limits_{i = 1}^{{N_{s} }} {\sum\limits_{j = 1}^{{iK_{0} }} {P\left( {i,j} \right) \cdot j \cdot Q_{TR100T} } } + \sum\limits_{i = 1}^{{N_{s} }} {\sum\limits_{{i = iK_{0} + 1}}^{{N_{t} }} {P\left( {i,j} \right) \cdot iK_{0} \cdot Q_{TR100T} } } = 608.29 \cdot {1}0^{{4}} \;{\text{m}}^{{3}} {\text{/a}}$$
④ 4 m^3^ hydraulic backhoe + 60t dump truck:
  $$Q_{{4m^{3} }} = \sum\limits_{i = 1}^{{N_{s} }} {\sum\limits_{j = 1}^{{iK_{0} }} {P\left( {i,j} \right) \cdot j \cdot Q_{60t} } } + \sum\limits_{i = 1}^{{N_{s} }} {\sum\limits_{{i = iK_{0} + 1}}^{{N_{t} }} {P\left( {i,j} \right) \cdot iK_{0} \cdot Q_{60t} } } = 10313.50 \cdot {1}0^{{4}} \;{\text{m}}^{{3}} {\text{/a}}$$

The daily operation efficiency of different types of shovels can be calculated using Eq. (11).

① $$\tilde{Q}_{WK - 35} = \frac{1954.16}{{6 \times 330}} \times 10000 = 9869.50\;{\text{m}}^{{3}} {\text{/d;}}$$
② $$\tilde{Q}_{WK - 10B} = \frac{993.21}{{4 \times 330}} \times 10000 = 7524.32\;{\text{m}}^{{3}} {\text{/d;}}$$
③ $$\tilde{Q}_{WK - 12} = \frac{608.29}{{2 \times 330}} \times 10000 = 9216.52\;{\text{m}}^{{3}} {\text{/d;}}$$
④ $$\tilde{Q}_{{4m^{3} }} = \frac{10313.50}{{106 \times 330}} \times 10000 = 2948.40\;{\text{m}}^{{3}} {\text{/d}}{.}$$

#### Block model size

**Figure 7:**
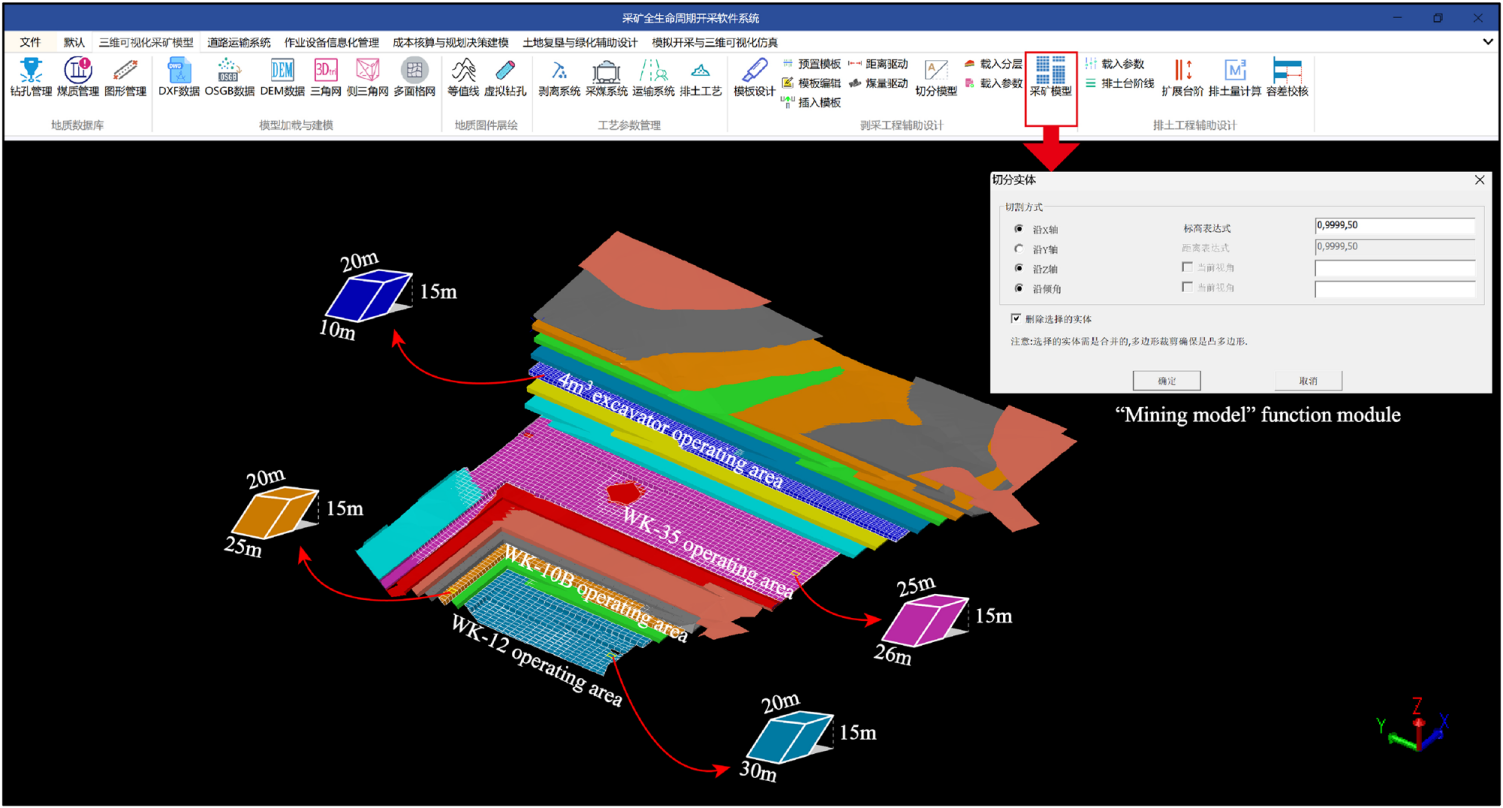
The self-developed software system and the generation of the ‘Mining model’.

Based on the mining parameters outlined in section "Mining parameter" and the daily operation efficiency of each shovel model calculated in section "Shovel-truck’s operation efficiency", the *X*-axis size of the block model for each shovel's working area are calculated using Eq. (11) as follows:

① One of the working areas of the shovel WK-35 is at the level of 565 m to 580 m. The *X*-axis size of the block model for this specific bench is: $$\overset{\lower0.5em\hbox{$\smash{\scriptscriptstyle\frown}$}}{X}_{WK - 35} = \frac{{\tilde{Q}_{WK - 35} }}{{\overset{\lower0.5em\hbox{$\smash{\scriptscriptstyle\frown}$}}{Y}_{WK - 35} \cdot \overset{\lower0.5em\hbox{$\smash{\scriptscriptstyle\frown}$}}{Z} }} = \frac{9869.50}{{25 \times 15}} = 26$$ m;
② One of the working areas of the shovel WK-10B is at the level of 505 m to 520 m. The *X*-axis size of the block model for this specific bench is: $$\overset{\lower0.5em\hbox{$\smash{\scriptscriptstyle\frown}$}}{X}_{WK - 10B} = \frac{{\tilde{Q}_{WK - 10B} }}{{\overset{\lower0.5em\hbox{$\smash{\scriptscriptstyle\frown}$}}{Y}_{WK - 10B} \cdot \overset{\lower0.5em\hbox{$\smash{\scriptscriptstyle\frown}$}}{Z} }} = \frac{7524.32}{{20 \times 15}} = 25$$ m;
③ One of the working areas of the shovel WK-12 is at the level of 490 m to 505 m. The *X*-axis size of the block model for this specific bench is: $$\overset{\lower0.5em\hbox{$\smash{\scriptscriptstyle\frown}$}}{X}_{WK - 12} = \frac{{\tilde{Q}_{WK - 12} }}{{\overset{\lower0.5em\hbox{$\smash{\scriptscriptstyle\frown}$}}{Y}_{WK - 12} \cdot \overset{\lower0.5em\hbox{$\smash{\scriptscriptstyle\frown}$}}{Z} }} = \frac{9216.52}{{20 \times 15}} = 30$$ m;
④ One of the working areas of the 4 m^3^ hydraulic backhoes is at the level of 590 m to 610 m. The *X*-axis size of the block model for this specific bench is: $$\overset{\lower0.5em\hbox{$\smash{\scriptscriptstyle\frown}$}}{X}_{{4m^{3} }} = \frac{{\tilde{Q}_{{4m^{3} }} }}{{\overset{\lower0.5em\hbox{$\smash{\scriptscriptstyle\frown}$}}{Y}_{{4m^{3} }} \cdot \overset{\lower0.5em\hbox{$\smash{\scriptscriptstyle\frown}$}}{Z} }} = \frac{2948.40}{{10 \times 15}} = 20$$ m;

The "Mining model" function module of the self-developed "Life Cycle Mining Software System" was developed using the C++ programming language. The module successfully realizes the construction of the block model for this study, as depicted in Fig. 7.

### Accuracy comparative analysis

**Figure 8:**
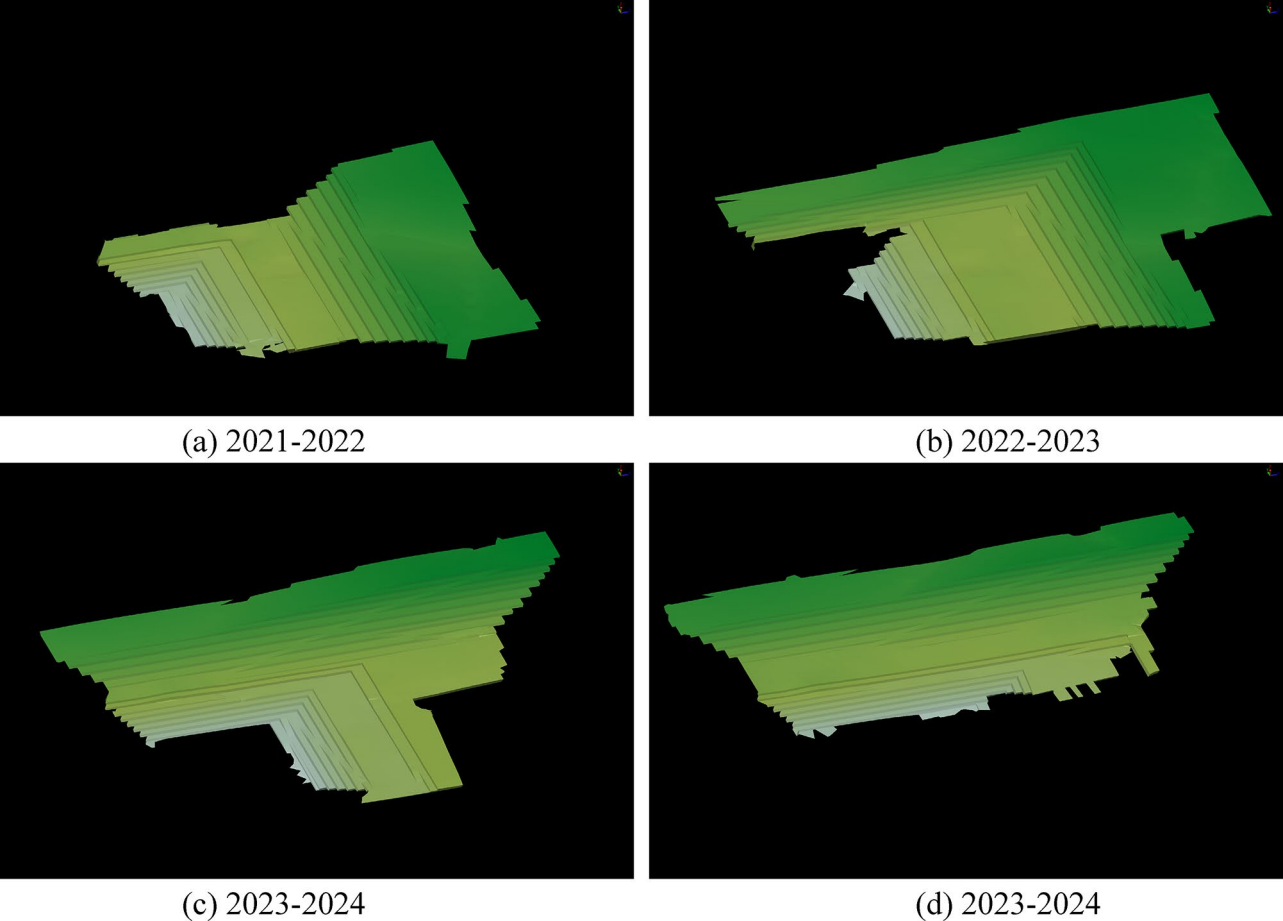
Closed shell model of 2021–2025 mining and stripping project in Baorixile open-pit mine.

**Figure 9:**
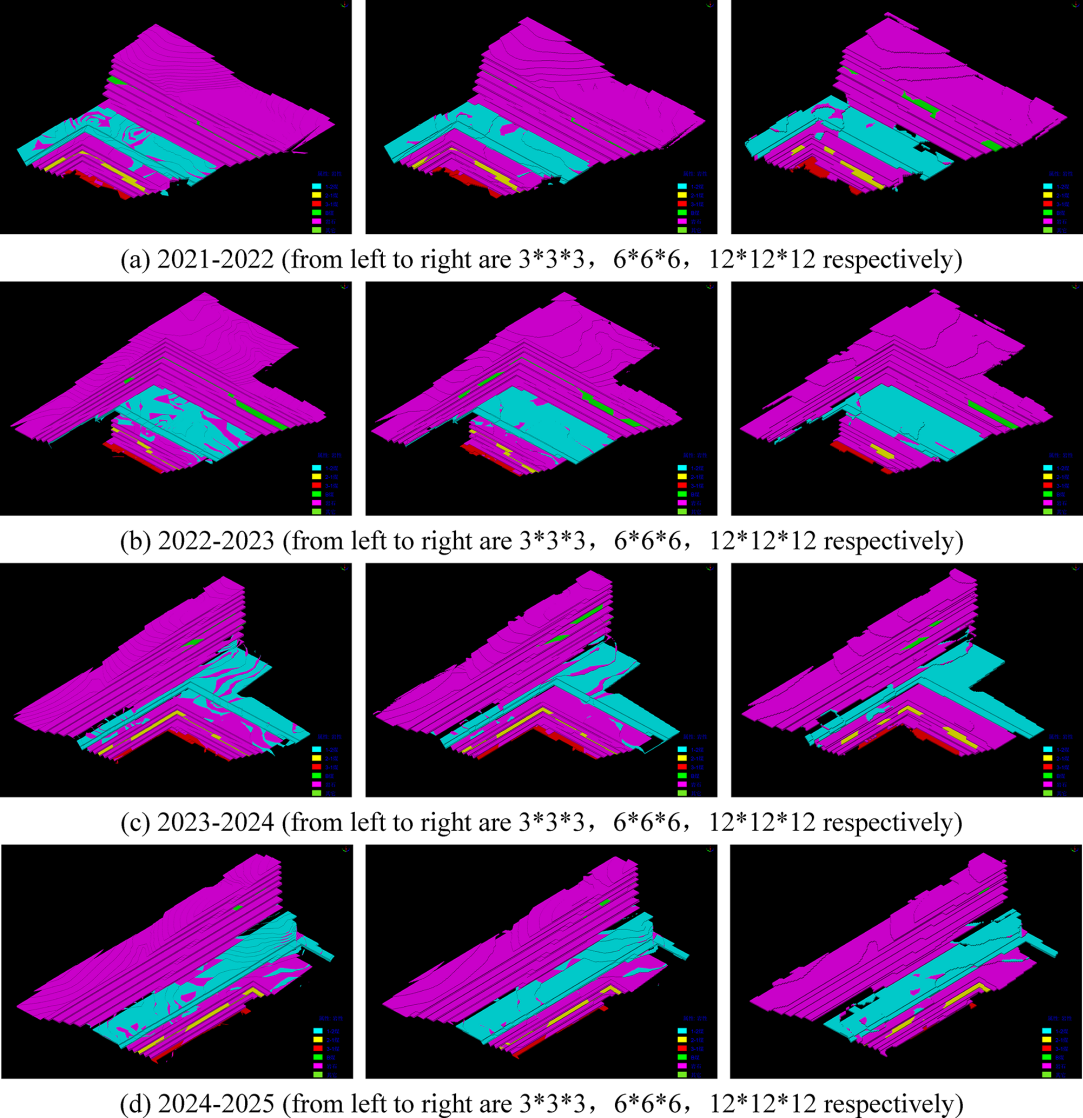
Block models of mining engineering with different specifications in 2021–2025.

The closed shell model is a geometric representation defined by its surface or boundary, encompassing only the outer surface or boundary without considering the specific shape of the interior set. The solid model provides a higher level of detail as it encompasses the internal structure and geometry. In comparison to the block model, the solid model offers enhanced computational accuracy; however, it comes with a trade-off of significantly slower efficiency. If the block were to be divided based on mining process parameters and equipment efficiency using the solid model as a foundation, the limited computing power of the computer would hinder the completion of this operation. In the case of layered deposits, there is no disparity in volume accuracy between the closed shell model and the solid model. Additionally, the computer exhibits fast operation speed when performing segmentation on the shell model. Consequently, this study opts for the closed shell model to construct the “Mining model”. To assess the superiority of the proposed method in terms of volume accuracy, a comparative analysis was conducted using the four-year mining and stripping engineering models of Baorixile open-pit mine. The selected time periods for analysis encompassed the years 2021 to 2022, 2022 to 2023, 2023 to 2024, and 2023 to 2024. This study employed these specific models to facilitate a comprehensive evaluation. Figure 8 illustrates the closed shell model depicting the mining and stripping engineering operations over a span of four years. The annual stripping engineering model is constructed separately using block configurations of 3 * 3 * 3, 6 * 6 * 6, 12 * 12 * 12, as shown in Fig. 9.

Table 5 illustrates the annual quantities of Coal B, Coal 1^2^, Coal 2^1^, and Coal 3^1^, as well as the total coal quantity, total volume, and error between the closed shell model and the block models, within the closed shell model and the corresponding 3 * 3 * 3, 6 * 6 * 6, 12 * 12 * 12 three different specifications of the block model of the mining and stripping project for the four years production plans.

**Table 5:** Precision comparison analysis table of closed shell model and block models.

Model		Type	2021–2022	2022–2023	2023–2024	2024–2025
Closed shell block model		Coal B (× 10^4^ t)	671.03	740.59	111.36	26.06
		Coal 1^2^ (× 10^4^ t)	1687.17	2472.32	2154.03	2230.13
		Coal 2^1^ (× 10^4^ t)	327.91	443.93	524.59	523.41
		Coal 3^1^ (× 10^4^ t)	288.95	370.44	422.61	294.38
		Total coal weight (× 10^4^ t)	2975.06	4027.28	3212.58	3073.99
		Total volume (× 10^4^ m^3^)	15,439.96	21,503.69	13,832.54	13,476.78
Traditional regular block model	3 * 3 * 3	Coal B (× 10^4^ t)	667.67	720.98	111.23	23.54
		Coal 1^2^ (× 10^4^ t)	1690.06	2457.53	2199.36	2240.21
		Coal 2^1^ (× 10^4^ t)	336.03	451.71	528.30	527.99
		Coal 3^1^ (× 10^4^ t)	288.99	376.93	429.57	297.05
		Total coal weight (× 10^4^ t)	2982.75	4007.16	3268.45	3088.78
		Total volume (× 10^4^ m^3^)	15,493.19	21,637.85	13,851.31	13,507.72
	6 * 6 * 6	Coal B (× 10^4^ t)	657.39	761.48	112.33	25.43
		Coal 1^2^ (× 10^4^ t)	1683.14	2449.95	2207.76	2264.79
		Coal 2^1^ (× 10^4^ t)	334.25	444.67	516.50	532.62
		Coal 3^1^ (× 10^4^ t)	279.07	378.70	436.50	321.79
		Total coal weight (× 10^4^ t)	2953.85	4034.79	3273.09	3144.63
		Total volume (× 10^4^ m^3^)	15,284.31	21,824.79	13,991.01	13,810.98
	12 * 12 * 12	Coal B (× 10^4^ t)	460.03	687.54	103.83	31.87
		Coal 1^2^ (× 10^4^ t)	1703.61	2457.09	2308.76	2187.49
		Coal 2^1^ (× 10^4^ t)	304.08	467.44	460.43	453.81
		Coal 3^1^ (× 10^4^ t)	286.44	374.44	374.64	177.80
		Total coal weight (× 10^4^ t)	2754.16	3986.51	3247.66	2850.97
		Total volume (× 10^4^ m^3^)	15,273.27	21,967.20	13,860.98	13,625.80
Error (%)	3 * 3 * 3	Coal B	0.5013	2.6471	0.1174	9.6828
		Coal 1^2^	0.1717	0.5982	2.1046	0.4516
		Coal 2^1^	2.4778	1.7520	0.7069	0.8744
		Coal 3^1^	0.0111	1.7519	1.6465	0.9083
		Total coal weight	0.2585	0.4997	1.7391	0.4814
		Total volume	0.3447	0.6239	0.1357	0.2296
	6 * 6 * 6	Coal B	2.0322	2.8207	0.8698	2.4247
		Coal 1^2^	0.2389	0.9047	2.4946	1.5538
		Coal 2^1^	1.9334	0.1653	1.5410	1.7589
		Coal 3^1^	3.4195	2.2279	3.2866	9.3134
		Total coal weight	0.7129	0.1865	1.8835	2.2981
		Total volume	1.0081	1.4932	1.1456	2.4798
	12 * 12 * 12	Coal B	31.4446	7.1633	6.7579	22.2816
		Coal 1^2^	0.9744	0.6158	7.1835	1.9123
		Coal 2^1^	7.2666	5.2958	12.2304	13.2965
		Coal 3^1^	0.8702	1.0781	11.3518	39.6023
		Total coal weight	7.4252	1.0124	1.0918	7.2549
		Total volume	1.0796	2.1555	0.2056	1.1057

**Figure 10:**
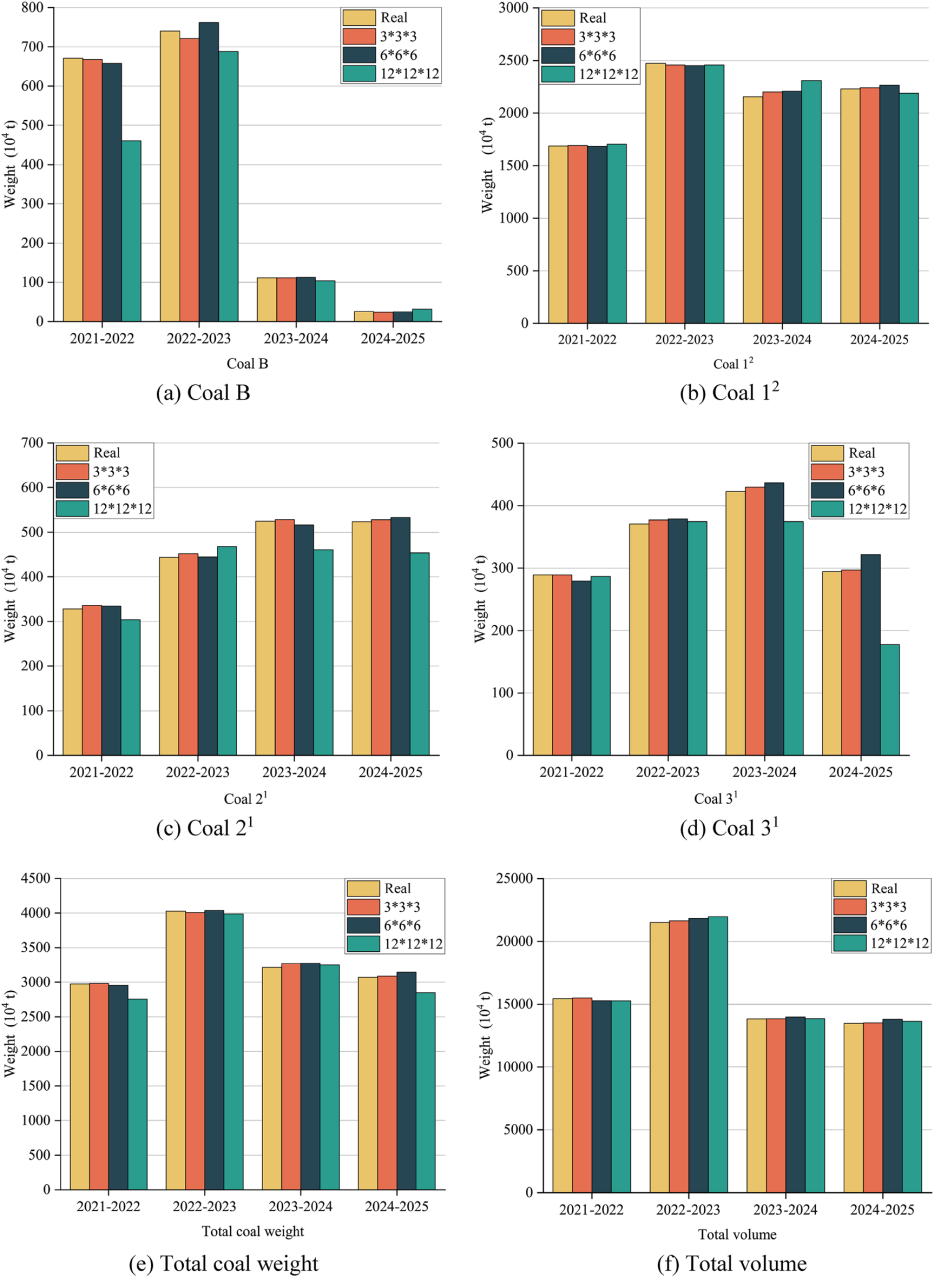
The comparison of coal quantity of each layer, total coal quantity and total volume between the closed shell model and the corresponding block model for 4 years.

**Figure 11:**
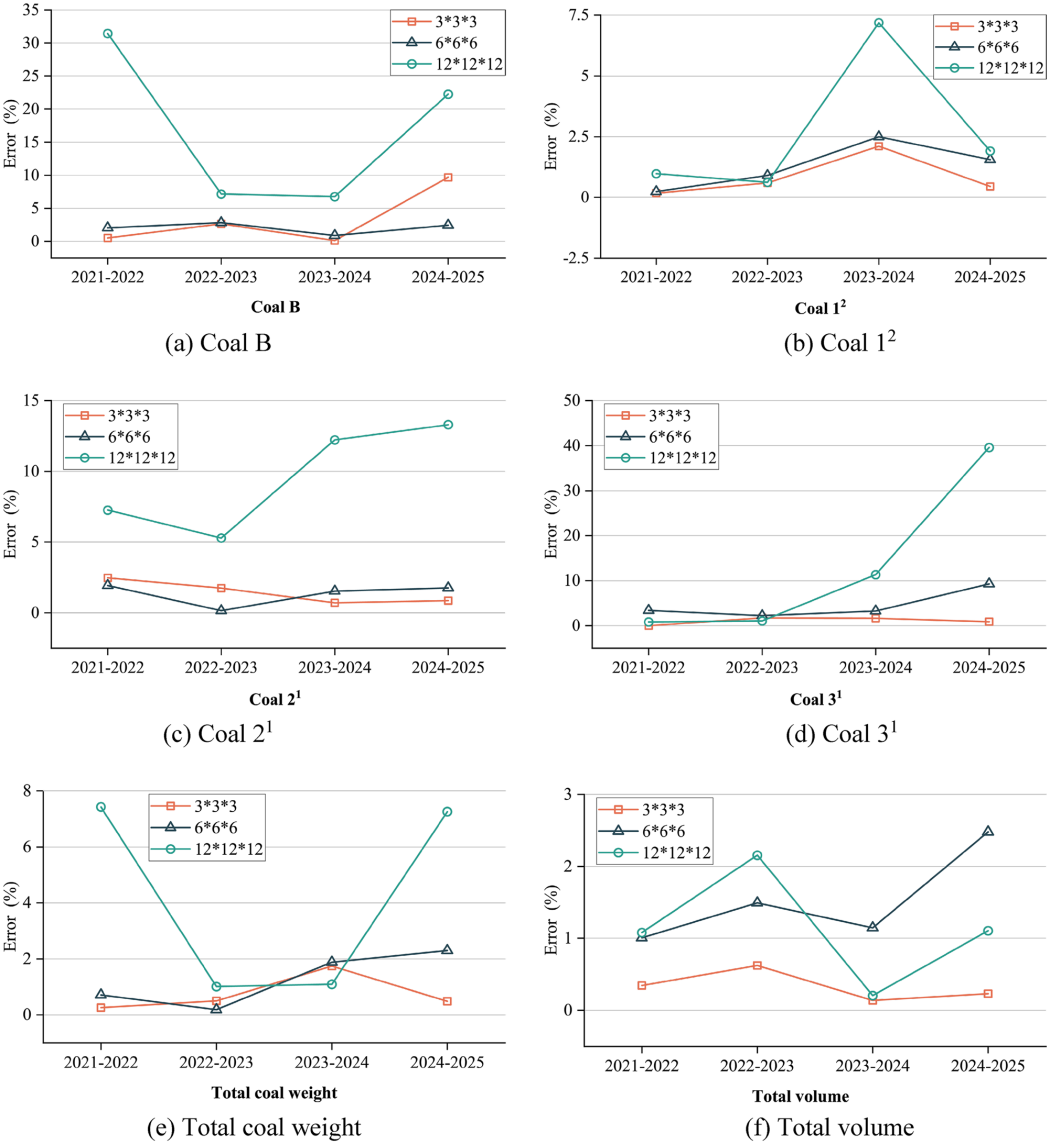
The accuracy error of 4 years of different specifications of mining and stripping engineering block model.

To provide a clearer representation of the engineering quantity errors between the three different specifications of the block model and the closed shell model at each stage, two comparison diagrams (refer to Figs. 10 and 11) are presented. These diagrams aim to enhance the understanding and visual depiction of the variations in engineering quantity. After conducting a comprehensive comparison, it is evident that the 12 * 12 * 12 block model generally exhibits the highest error, while the 3 * 3 * 3 block model demonstrates the lowest error in general. Nevertheless, it is not necessarily that as the regular block model size decreases, the accuracy tends to improve. For instance, the 6 * 6 * 6 block model for the 2024–2025 period in Fig. 10a and for the 2021–2022 and 2022–2023 periods in Fig. 10c, exhibit higher accuracy compared to the 3 * 3 * 3 block model. The 12 * 12 * 12 block model exhibit higher accuracy compared to the 3 * 3 * 3 block model for the 2023–2024 period in Fig. 10e. In the design of the production plan for the next few years, the open-pit mine will not always carry out the experiment of optimal block size selection. Therefore, the presence of interference factors resulting from human subjective consciousness continues to exert a significant influence on the accuracy. Upon error superposition, the block specification with the highest error in the 2021–2022 period, as depicted in Fig. 11e, is 12 × 12 × 12, with a maximum error reaching 7.4252%. Similarly, in Fig. 11f, the block specification with the highest error is 6 × 6 × 6, with a maximum error of 2.4798%. This highlights the challenge in selecting the optimal block size when utilizing a regular block model for constructing a three-dimensional geological body. Simultaneously, the findings also demonstrate that the block division method proposed in this study yields the highest level of accuracy in terms of volume precision. The method introduced in this study effectively mitigates volume errors associated with utilizing a regular block model to populate the three-dimensional geological model for production planning design. Moreover, it ensures the regularity of mining benches, aligning more closely with the practical construction conditions in open-pit mines.

## Discussions

In this study, the aim was to enhance the accuracy of the foundational model for production planning by expanding the application of the open-pit mine three-dimensional geological model. To achieve this, a departure from the conventional approach of employing a regular block model to populate the solid model was pursued. To achieve this, a departure from the conventional approach of employing a regular block model to populate the solid model was pursued. The closed shell model is directly discretized into a block model while incorporating the constraints imposed by mining parameters and shovel-truck’s operation efficiency. This study serves as a foundation for our future research on attribute assignment of the mining model, block model migration order, and migration path prediction. Our overarching objective is to develop a comprehensive optimization method for production planning throughout the life cycle of open-pit mines, and to create a prototype system for decision-making in open-pit mine life cycle mining planning. It is noteworthy that in this study, the daily equipment operation efficiency is utilized to determine the X-axis size of the block. However, it is also possible to partition the blocks based on weekly or monthly equipment operation efficiency, enabling the creation of long-term production plans. Based on the diverse equipment types and their respective layout positions, the block size of the equipment's operational area can be determined. This approach aligns more closely with the actual operational conditions. Simultaneously, it significantly reduces the number of blocks, minimizes the computer's processing time, and enhances overall work efficiency. However, it is important to note that this study focuses specifically on the shovel-truck intermittent mining technology, and there remains a dearth of research and development regarding other mining technologies. Subsequent research endeavors will aim to build upon and expand the findings of this study, with the goal of further improvement and in-depth exploration.

## Conclusions

The following conclusions and comments can be made from the present study:

1. Based on the three-dimensional geological model of the closed shell type, the block model is generated considering mining parameters as constraints. Specifically, the bench height is employed as the *Z*-axis size of the block model, the mining belt width of is adopted as the *Y*-axis size of the block model, and the bench slope angle serves as the inclination angle of the block model.
2. Considering the random fluctuation of the number of equipment, compared with the method of calculating equipment efficiency and output by average index, the results of equipment operation efficiency obtained by probability analysis method are more in line with the actual situation. In this study, the daily equipment outburst performance of each type of excavator is calculated based on the maximum probability of simultaneous operation of self-operated and outsourced units. This calculation aids in determining the *X*-axis size of the block model in the operational area of different types of excavators.
3. In this paper, using Baorixile open-pit mine as a case study, a ‘Mining model’ function module is developed within the self-developed system. A closed shell block model of the mining and stripping project at the open-pit mine for a four-year period from 2021 to 2025 was created. The regular block models of 3 * 3 * 3, 6 * 6 * 6, and 12 * 12 * 12 were selected and compared to the block model used in this study. This highlights the challenge in selecting the optimal block size when utilizing a regular block model for constructing a three-dimensional geological body. Simultaneously, the findings also demonstrate that the block division method proposed in this study yields the highest level of accuracy in terms of volume precision. The method introduced in this study effectively mitigates volume errors associated with utilizing a regular block model to populate the three-dimensional geological model for production planning design. Moreover, it ensures the regularity of mining benches, aligning more closely with the practical construction conditions in open-pit mines.

## Data Availability

The data used and analysed during the current study available from the corresponding author on reasonable request.
