# Author Correction: Research on the method of determining the block size for an open-pit mine integrating mining parameters and shovel-truck’s operation efficiency

**DOI:** 10.1038/s41598-024-62919-x

**Published:** 2024-05-31

**Authors:** Weiqiang Guo, Guangwei Liu, Jiaming Li, Senlin Chai, Shupeng Guo

**Affiliations:** 1 School of Mining, Liaoning Technical University, Fuxin, 123000 China; 2 School of Economics & Management, Yancheng Institute of Technology, Yancheng, 224051 China; 3 State Grid Energy Hami Coal and Electricity Co., Ltd., Dananhu No. 2 Mine, Hami, 839000 China

Correction to: *Scientific Reports* 10.1038/s41598-024-52815-9, published online 02 May 2024

The original version of this Article contained an error in the Acknowledgements section, where the grant number for the National Natural Science Foundation of China was incorrect. Consequently,

“This research was supported by the National Natural Science Foundation of China (51974144), the Youth Fund of the National Natural Science Foundation of China (52204158), 'Jie Bang Gua Shuai' (Take the Lead) of the Key Scientific and Technological Project for Liaoning Province (2021JH1/10400011), Discipline Innovation Team of Liaoning Technical University (LNTU20TD-07), and School level scientific research project of Yancheng Institute of Technology (xjr2020039).”

now reads:

“This research was supported by the National Natural Science Foundation of China (52374123), the Youth Fund of the National Natural Science Foundation of China (52204158), 'Jie Bang Gua Shuai' (Take the Lead) of the Key Scientific and Technological Project for Liaoning Province (2021JH1/10400011), Discipline Innovation Team of Liaoning Technical University (LNTU20TD-07), and School level scientific research project of Yancheng Institute of Technology (xjr2020039).”

The original Article has been corrected.

